# Bis[2-(2-pyridylmethyl­eneamino)benzene­sulfonato-κ^3^ *N*,*N*′,*O*]manganese(II) dihydrate

**DOI:** 10.1107/S1600536809030670

**Published:** 2009-08-08

**Authors:** Cheng-Qiang Zhao, Miao Ou-Yang, Xue-Ren Huang, Yi-Min Jiang

**Affiliations:** a College of Chemistry and Chemical Engineering, Guangxi Normal University, Guilin, Guangxi 541004, People’s Republic of China; b Department of Chemistry and Life Science, Hechi University, Yizhou, Guangxi 546300, People’s Republic of China; c Leizhou No. 1 Middle School, Leizhou, Guangdong 524200, People’s Republic of China

## Abstract

The title complex, [Mn(C_12_H_9_N_2_O_3_S)_2_]·2H_2_O, is isotypic with the previously reported Zn^II^ and Cd^II^ species. The complex was prepared by the reaction of the potassium salt of 2-(2-pyridylmethyl­eneamino)benzene­sulfonic acid with MnCl_2_·6H_2_O in methanol. The complex displays twofold symmetry, with the ligands coordinated in a tridentate meridional-like arrangement through pyridyl N, imine N, and sulfonate O atoms. The metal center has a strongly distorted octa­hedral coordination geometry. The uncoordin­ated water mol­ecules and the complexes participate in a hydrogen-bonding network, forming a two-dimensional structure parallel to the *ab* plane.

## Related literature

For the synthesis of the ligand, see: Casella & Gullotti (1986 ▶). For the structures of the Zn^II^ and Cd^II^ analogues, see: Cai *et al.* (2008 ▶); Ou-Yang *et al.* (2008 ▶). For related Schiff bases complexes, see: Correia *et al.* (2003 ▶); Li *et al.* (2007 ▶, 2008 ▶); Ou-Yang *et al.* (2009 ▶); Zhang *et al.* (2005 ▶).
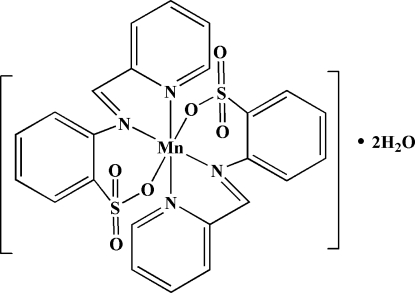


## Experimental

### 

#### Crystal data

[Mn(C_12_H_9_N_2_O_3_S)_2_]·2H_2_O
*M* *_r_* = 613.52
Orthorhombic, 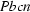

*a* = 20.041 (10) Å
*b* = 7.918 (4) Å
*c* = 16.409 (8) Å
*V* = 2604 (2) Å^3^
*Z* = 4
Mo *K*α radiation
μ = 0.72 mm^−1^
*T* = 296 K
0.49 × 0.34 × 0.21 mm

#### Data collection

SMART APEX CCD diffractometer
Absorption correction: none
13313 measured reflections
2320 independent reflections
1950 reflections with *I* > 2σ(*I*)
*R*_int_ = 0.028

#### Refinement

*R*[*F* ^2^ > 2σ(*F* ^2^)] = 0.032
*wR*(*F* ^2^) = 0.092
*S* = 1.01
2320 reflections
177 parameters
H-atom parameters constrained
Δρ_max_ = 0.47 e Å^−3^
Δρ_min_ = −0.41 e Å^−3^

### 

Data collection: *SMART* (Bruker, 2004 ▶); cell refinement: *SAINT* (Bruker, 2004 ▶); data reduction: *SAINT*; program(s) used to solve structure: *SHELXS97* (Sheldrick, 2008 ▶); program(s) used to refine structure: *SHELXL97* (Sheldrick, 2008 ▶); molecular graphics: *SHELXTL* (Sheldrick, 2008 ▶); software used to prepare material for publication: *SHELXTL*.

## Supplementary Material

Crystal structure: contains datablocks I, global. DOI: 10.1107/S1600536809030670/bh2236sup1.cif

Structure factors: contains datablocks I. DOI: 10.1107/S1600536809030670/bh2236Isup2.hkl

Additional supplementary materials:  crystallographic information; 3D view; checkCIF report

## Figures and Tables

**Table 1:** Hydrogen-bond geometry (Å, °)

*D*—H⋯*A*	*D*—H	H⋯*A*	*D*⋯*A*	*D*—H⋯*A*
O4—H1*W*⋯O3^i^	0.85	2.14	2.993 (3)	179
O4—H2*W*⋯O2	0.84	2.11	2.866 (3)	151

Symmetry code: (i) 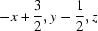.

**Fig. 1.**
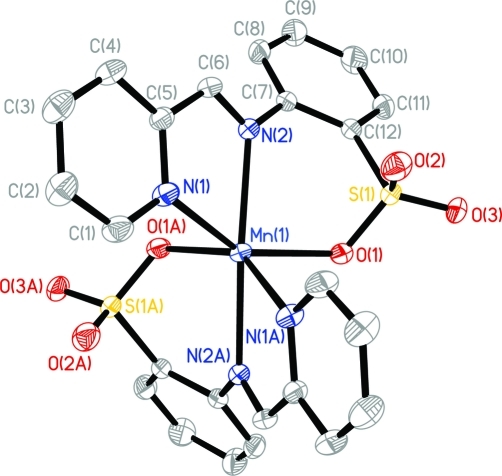
The molecular structure of the title complex, showing the atom-numbering scheme. The water molecule and all H atoms have been omitted for clarity. Symmetry code for 'A' labelled atoms: 2 - x, y, 1/2 - z.

**Fig. 2.**
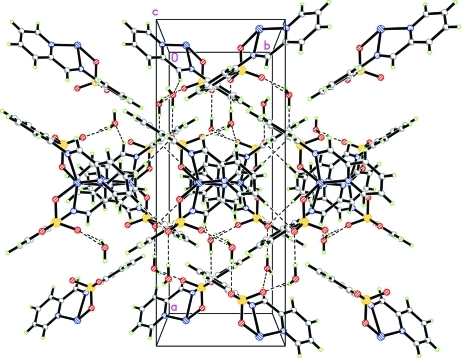
Packing of the title complex, showing the two-dimensional network in the ab plane, linked via hydrogen bonds (dashed lines).

## supplementary crystallographic information

### Comment

In the past decades, Schiff-base complexes containing sulfonate have received much attention owing to the diverse coordination modes and bridging ability (Zhang *et al.*, 2005; Li *et al.*, 2007; Ou-Yang *et al.*, 2009). Herein, we prepared a mononuclear Mn(II) complex, which is isostructural with [Zn(Paba)_2_].2 H_2_O and [Cd(Paba)_2_].2 H_2_O, whose structures were described in details (Cai *et al.*, 2008; Ou-Yang *et al.*, 2008). The six-coordinated Mn^II^ ion lies on a twofold axis, and two deprotonated PabaH anions coordinate to Mn in a *meridional* arrangement as *N*,*N*',*O*-tridentate donor ligands. The coordination mode of the complex is similar to that observed in other complexes with *N*,*N*',*O*-tridentate donor ligands (Li *et al.*, 2007, 2008; Correia *et al.*, 2003).

The O—H donor groups from the lattice water molecules and the SO acceptor groups of the Paba ligands participate in the hydrogen bonding through which the complex completes a two-dimensional network parallel to the *ab* plane (Fig. 2).

### Experimental

The potassium salt of 2-(2-pyridylmethyleneamino)benzenesulfonic acid (PabaK) was synthesized according to the literature methods (Casella & Gullotti, 1986). The ligand PabaK (1 mmol, 0.30 g) was dissolved in methanol (10 ml). To this solution, MnCl_2_ 6 H_2_O (0.5 mmol, 0.12 g) was added, and the resulting mixture was stirred at 333 K for 4 h. Then the mixture was filtrated and the filtrate was left to stand at room temperature. Yellow crystals suitable for X-ray diffraction were obtained after a week in a yield of 55%. Elemental analysis: found C 46.87, H 4.04, N 9.05, S 10.42%; calc. C 46.94, H 3.59, N 9.13, S 10.43%.

### Refinement

C-bonded H atoms were positioned geometrically with C—H distances of 0.93 Å, and treated as riding atoms, with *U*_iso_(H) = 1.2 *U*_eq_(carrier C). Water H atoms were placed in fixed positions and assigned *U*_iso_ values of 1.5 *U*_eq_ of the water O atom.

### Crystal data

**Table:**

[Mn(C_12_H_9_N_2_O_3_S)_2_]·2H_2_O	*F*(000) = 1260
*M**_r_* = 613.52	*D*_x_ = 1.565 Mg m^−^^3^
Orthorhombic, *P**b**c**n*	Mo *K*α radiation, λ = 0.71073 Å
Hall symbol: -P 2n 2ab	Cell parameters from 4677 reflections
*a* = 20.041 (10) Å	θ = 2.5–28.5°
*b* = 7.918 (4) Å	µ = 0.72 mm^−^^1^
*c* = 16.409 (8) Å	*T* = 296 K
*V* = 2604 (2) Å^3^	Block, yellow
*Z* = 4	0.49 × 0.34 × 0.21 mm

### Data collection

**Table:**

SMART APEX CCD diffractometer	1950 reflections with *I* > 2σ(*I*)
Radiation source: fine-focus sealed tube	*R*_int_ = 0.028
graphite	θ_max_ = 25.1°, θ_min_ = 2.5°
φ and ω scans	*h* = −21→23
13313 measured reflections	*k* = −9→9
2320 independent reflections	*l* = −19→19

### Refinement

**Table:**

Refinement on *F*^2^	Primary atom site location: structure-invariant direct methods
Least-squares matrix: full	Secondary atom site location: difference Fourier map
*R*[*F*^2^ > 2σ(*F*^2^)] = 0.032	Hydrogen site location: inferred from neighbouring sites
*wR*(*F*^2^) = 0.092	H-atom parameters constrained
*S* = 1.01	*w* = 1/[σ^2^(*F*_o_^2^) + (0.0512*P*)^2^ + 1.5899*P*] where *P* = (*F*_o_^2^ + 2*F*_c_^2^)/3
2320 reflections	(Δ/σ)_max_ < 0.001
177 parameters	Δρ_max_ = 0.47 e Å^−^^3^
0 restraints	Δρ_min_ = −0.41 e Å^−^^3^

### Fractional atomic coordinates and isotropic or equivalent isotropic displacement parameters (Å^2^)

**Table:**

	*x*	*y*	*z*	*U*_iso_*/*U*_eq_	
Mn1	1.0000	0.18060 (6)	0.2500	0.02835 (15)	
S1	0.87380 (3)	0.32706 (7)	0.33859 (3)	0.03333 (17)	
O1	0.94725 (8)	0.3323 (2)	0.33645 (9)	0.0381 (4)	
O2	0.84921 (9)	0.1575 (2)	0.32866 (10)	0.0508 (5)	
O3	0.84826 (8)	0.4155 (2)	0.40879 (9)	0.0466 (4)	
N1	0.99221 (9)	−0.0275 (2)	0.15711 (11)	0.0352 (4)	
N2	0.90694 (8)	0.2347 (2)	0.16781 (10)	0.0303 (4)	
C1	1.03597 (13)	−0.1521 (3)	0.14852 (14)	0.0444 (6)	
H1	1.0708	−0.1601	0.1858	0.053*	
C2	1.03222 (15)	−0.2704 (3)	0.08686 (15)	0.0495 (6)	
H2	1.0641	−0.3553	0.0826	0.059*	
C3	0.98074 (14)	−0.2607 (3)	0.03207 (16)	0.0503 (6)	
H3	0.9767	−0.3399	−0.0095	0.060*	
C4	0.93496 (13)	−0.1310 (3)	0.03971 (14)	0.0440 (6)	
H4	0.8998	−0.1210	0.0031	0.053*	
C5	0.94222 (11)	−0.0165 (3)	0.10256 (12)	0.0329 (5)	
C6	0.89698 (11)	0.1272 (3)	0.11156 (13)	0.0360 (5)	
H6	0.8610	0.1400	0.0762	0.043*	
C7	0.86610 (10)	0.3806 (3)	0.17345 (12)	0.0321 (5)	
C8	0.84533 (12)	0.4702 (3)	0.10524 (14)	0.0440 (6)	
H8	0.8560	0.4311	0.0534	0.053*	
C9	0.80902 (13)	0.6167 (4)	0.11399 (15)	0.0536 (7)	
H9	0.7950	0.6754	0.0680	0.064*	
C10	0.79326 (14)	0.6770 (4)	0.19056 (17)	0.0542 (7)	
H10	0.7695	0.7772	0.1960	0.065*	
C11	0.81279 (12)	0.5887 (3)	0.25907 (14)	0.0432 (6)	
H11	0.8014	0.6283	0.3106	0.052*	
C12	0.84920 (10)	0.4420 (3)	0.25118 (11)	0.0319 (5)	
O4	0.79877 (12)	−0.0889 (4)	0.44087 (15)	0.1140 (12)	
H1W	0.7570	−0.0869	0.4320	0.171*	
H2W	0.8166	−0.0498	0.3988	0.171*	

### Atomic displacement parameters (Å^2^)

**Table:**

	*U*^11^	*U*^22^	*U*^33^	*U*^12^	*U*^13^	*U*^23^
Mn1	0.0279 (3)	0.0336 (3)	0.0235 (2)	0.000	−0.00482 (17)	0.000
S1	0.0326 (3)	0.0437 (3)	0.0236 (3)	0.0002 (2)	0.0001 (2)	0.0008 (2)
O1	0.0316 (8)	0.0540 (10)	0.0287 (8)	0.0050 (7)	−0.0044 (6)	−0.0070 (7)
O2	0.0606 (12)	0.0486 (11)	0.0431 (10)	−0.0120 (9)	−0.0038 (8)	0.0083 (8)
O3	0.0434 (9)	0.0699 (12)	0.0267 (8)	0.0085 (8)	0.0051 (7)	−0.0043 (8)
N1	0.0409 (10)	0.0350 (10)	0.0297 (9)	0.0024 (8)	−0.0067 (8)	−0.0015 (8)
N2	0.0280 (9)	0.0384 (10)	0.0245 (8)	0.0003 (8)	−0.0007 (7)	−0.0004 (7)
C1	0.0503 (15)	0.0428 (14)	0.0401 (13)	0.0087 (12)	−0.0108 (11)	−0.0008 (10)
C2	0.0684 (17)	0.0356 (13)	0.0445 (14)	0.0116 (12)	−0.0024 (13)	−0.0030 (11)
C3	0.0721 (18)	0.0382 (13)	0.0406 (13)	0.0020 (13)	−0.0054 (13)	−0.0095 (11)
C4	0.0510 (15)	0.0466 (14)	0.0345 (12)	−0.0044 (11)	−0.0110 (11)	−0.0076 (10)
C5	0.0350 (11)	0.0369 (12)	0.0269 (10)	−0.0033 (9)	−0.0028 (9)	−0.0003 (9)
C6	0.0318 (11)	0.0488 (13)	0.0273 (11)	−0.0008 (10)	−0.0067 (9)	−0.0017 (10)
C7	0.0243 (11)	0.0435 (13)	0.0284 (10)	0.0018 (9)	−0.0006 (8)	0.0017 (9)
C8	0.0398 (13)	0.0621 (16)	0.0302 (11)	0.0098 (12)	0.0019 (10)	0.0066 (11)
C9	0.0495 (15)	0.0690 (18)	0.0421 (14)	0.0200 (14)	0.0001 (12)	0.0159 (13)
C10	0.0497 (16)	0.0556 (17)	0.0574 (16)	0.0213 (13)	0.0015 (13)	0.0089 (13)
C11	0.0380 (13)	0.0517 (15)	0.0398 (13)	0.0107 (11)	0.0045 (10)	−0.0024 (11)
C12	0.0257 (11)	0.0415 (12)	0.0284 (11)	−0.0003 (9)	0.0002 (8)	0.0014 (9)
O4	0.0640 (15)	0.182 (3)	0.0956 (19)	−0.0353 (17)	−0.0233 (13)	0.075 (2)

### Geometric parameters (Å, °)

**Table:**

Mn1—O1	2.1382 (16)	C3—C4	1.383 (4)
Mn1—O1^i^	2.1382 (16)	C3—H3	0.9300
Mn1—N1	2.250 (2)	C4—C5	1.381 (3)
Mn1—N1^i^	2.250 (2)	C4—H4	0.9300
Mn1—N2^i^	2.3412 (19)	C5—C6	1.463 (3)
Mn1—N2	2.3412 (19)	C6—H6	0.9300
S1—O2	1.4395 (19)	C7—C8	1.389 (3)
S1—O3	1.4420 (17)	C7—C12	1.406 (3)
S1—O1	1.4729 (18)	C8—C9	1.376 (4)
S1—C12	1.769 (2)	C8—H8	0.9300
N1—C1	1.327 (3)	C9—C10	1.381 (4)
N1—C5	1.346 (3)	C9—H9	0.9300
N2—C6	1.271 (3)	C10—C11	1.380 (3)
N2—C7	1.418 (3)	C10—H10	0.9300
C1—C2	1.381 (3)	C11—C12	1.378 (3)
C1—H1	0.9300	C11—H11	0.9300
C2—C3	1.371 (4)	O4—H1W	0.8495
C2—H2	0.9300	O4—H2W	0.8364
			
O1—Mn1—O1^i^	111.67 (10)	C1—C2—H2	120.6
O1—Mn1—N1	145.73 (6)	C2—C3—C4	118.8 (2)
O1^i^—Mn1—N1	89.79 (7)	C2—C3—H3	120.6
O1—Mn1—N1^i^	89.79 (7)	C4—C3—H3	120.6
O1^i^—Mn1—N1^i^	145.73 (6)	C5—C4—C3	119.0 (2)
N1—Mn1—N1^i^	85.83 (10)	C5—C4—H4	120.5
O1—Mn1—N2^i^	84.77 (6)	C3—C4—H4	120.5
O1^i^—Mn1—N2^i^	83.42 (7)	N1—C5—C4	122.2 (2)
N1—Mn1—N2^i^	125.42 (7)	N1—C5—C6	116.40 (18)
N1^i^—Mn1—N2^i^	71.86 (7)	C4—C5—C6	121.4 (2)
O1—Mn1—N2	83.42 (6)	N2—C6—C5	119.80 (19)
O1^i^—Mn1—N2	84.77 (6)	N2—C6—H6	120.1
N1—Mn1—N2	71.86 (7)	C5—C6—H6	120.1
N1^i^—Mn1—N2	125.42 (7)	C8—C7—C12	118.8 (2)
N2^i^—Mn1—N2	158.90 (9)	C8—C7—N2	122.43 (19)
O2—S1—O3	114.95 (11)	C12—C7—N2	118.65 (18)
O2—S1—O1	111.44 (11)	C9—C8—C7	120.3 (2)
O3—S1—O1	111.11 (9)	C9—C8—H8	119.8
O2—S1—C12	107.02 (10)	C7—C8—H8	119.8
O3—S1—C12	107.40 (11)	C8—C9—C10	120.5 (2)
O1—S1—C12	104.18 (9)	C8—C9—H9	119.8
S1—O1—Mn1	119.62 (9)	C10—C9—H9	119.8
C1—N1—C5	117.98 (19)	C11—C10—C9	120.1 (2)
C1—N1—Mn1	124.77 (15)	C11—C10—H10	120.0
C5—N1—Mn1	117.04 (14)	C9—C10—H10	120.0
C6—N2—C7	120.13 (18)	C12—C11—C10	120.0 (2)
C6—N2—Mn1	114.88 (15)	C12—C11—H11	120.0
C7—N2—Mn1	124.83 (13)	C10—C11—H11	120.0
N1—C1—C2	123.1 (2)	C11—C12—C7	120.3 (2)
N1—C1—H1	118.5	C11—C12—S1	120.34 (16)
C2—C1—H1	118.5	C7—C12—S1	119.38 (17)
C3—C2—C1	118.9 (2)	H1W—O4—H2W	105.8
C3—C2—H2	120.6		
			
O2—S1—O1—Mn1	41.05 (13)	C2—C3—C4—C5	−0.5 (4)
O3—S1—O1—Mn1	170.64 (10)	C1—N1—C5—C4	0.9 (3)
C12—S1—O1—Mn1	−74.01 (13)	Mn1—N1—C5—C4	175.90 (17)
O1^i^—Mn1—O1—S1	115.14 (11)	C1—N1—C5—C6	−177.0 (2)
N1—Mn1—O1—S1	−10.02 (18)	Mn1—N1—C5—C6	−2.0 (2)
N1^i^—Mn1—O1—S1	−92.28 (11)	C3—C4—C5—N1	−0.5 (4)
N2^i^—Mn1—O1—S1	−164.08 (11)	C3—C4—C5—C6	177.4 (2)
N2—Mn1—O1—S1	33.44 (10)	C7—N2—C6—C5	175.63 (19)
O1—Mn1—N1—C1	−137.89 (18)	Mn1—N2—C6—C5	0.1 (3)
O1^i^—Mn1—N1—C1	91.6 (2)	N1—C5—C6—N2	1.3 (3)
N1^i^—Mn1—N1—C1	−54.42 (17)	C4—C5—C6—N2	−176.7 (2)
N2^i^—Mn1—N1—C1	9.8 (2)	C6—N2—C7—C8	−40.0 (3)
N2—Mn1—N1—C1	176.1 (2)	Mn1—N2—C7—C8	135.02 (19)
O1—Mn1—N1—C5	47.5 (2)	C6—N2—C7—C12	143.7 (2)
O1^i^—Mn1—N1—C5	−83.05 (16)	Mn1—N2—C7—C12	−41.3 (3)
N1^i^—Mn1—N1—C5	130.97 (18)	C12—C7—C8—C9	0.2 (4)
N2^i^—Mn1—N1—C5	−164.81 (14)	N2—C7—C8—C9	−176.1 (2)
N2—Mn1—N1—C5	1.52 (15)	C7—C8—C9—C10	0.5 (4)
O1—Mn1—N2—C6	−156.78 (16)	C8—C9—C10—C11	−1.3 (4)
O1^i^—Mn1—N2—C6	90.65 (16)	C9—C10—C11—C12	1.3 (4)
N1—Mn1—N2—C6	−0.84 (15)	C10—C11—C12—C7	−0.6 (4)
N1^i^—Mn1—N2—C6	−71.75 (17)	C10—C11—C12—S1	−179.4 (2)
N2^i^—Mn1—N2—C6	146.83 (16)	C8—C7—C12—C11	−0.1 (3)
O1—Mn1—N2—C7	27.95 (16)	N2—C7—C12—C11	176.3 (2)
O1^i^—Mn1—N2—C7	−84.62 (16)	C8—C7—C12—S1	178.73 (17)
N1—Mn1—N2—C7	−176.11 (17)	N2—C7—C12—S1	−4.8 (3)
N1^i^—Mn1—N2—C7	112.98 (16)	O2—S1—C12—C11	124.5 (2)
N2^i^—Mn1—N2—C7	−28.44 (15)	O3—S1—C12—C11	0.6 (2)
C5—N1—C1—C2	−0.4 (4)	O1—S1—C12—C11	−117.32 (19)
Mn1—N1—C1—C2	−174.95 (19)	O2—S1—C12—C7	−54.3 (2)
N1—C1—C2—C3	−0.6 (4)	O3—S1—C12—C7	−178.25 (17)
C1—C2—C3—C4	1.0 (4)	O1—S1—C12—C7	63.82 (19)

Symmetry codes: (i) −*x*+2, *y*, −*z*+1/2.

### Hydrogen-bond geometry (Å, °)

**Table:**

*D*—H···*A*	*D*—H	H···*A*	*D*···*A*	*D*—H···*A*
O4—H1W···O3^ii^	0.85	2.14	2.993 (3)	179
O4—H2W···O2	0.84	2.11	2.866 (3)	151

Symmetry codes: (ii) −*x*+3/2, *y*−1/2, *z*.
